# Ultrasound-assisted laser therapy for selective removal of melanoma cells

**DOI:** 10.3389/ebm.2024.10096

**Published:** 2024-08-07

**Authors:** Madhumithra Subramanian Karthikesh, Noraida Martinez-Rivera, Eduardo Rosa-Molinar, Xueding Wang, Xinmai Yang

**Affiliations:** ^1^ Bioengineering Graduate Program, University of Kansas, Lawrence, KS, United States; ^2^ Institute for Bioengineering Research, University of Kansas, Lawrence, KS, United States; ^3^ Microscopy and Analytical Imaging Laboratory, University of Kansas, Lawrence, KS, United States; ^4^ Department of Pharmacology and Toxicology, and Neuroscience, University of Kansas, Lawrence, KS, United States; ^5^ Department of Biomedical Engineering, University of Michigan, Ann Arbor, MI, United States; ^6^ Department of Mechanical Engineering, School of Engineering, University of Kansas, Lawrence, KS, United States

**Keywords:** ultrasound-assisted laser therapy, photo-mediated ultrasound therapy, melanoma treatment, cavitation, selective melanoma treatment

## Abstract

The current study explores the potential of ultrasound-assisted laser therapy (USaLT) to selectively destroy melanoma cells. The technology was tested on an *ex vivo* melanoma model, which was established by growing melanoma cells in chicken breast tissue. Ultrasound-only and laser-only treatments were used as control groups. USaLT was able to effectively destroy melanoma cells and selectively remove 66.41% of melanoma cells in the *ex vivo* tumor model when an ultrasound peak negative pressure of 2 MPa was concurrently applied with a laser fluence of 28 mJ/cm^2^ at 532 nm optical wavelength for 5 min. The therapeutic efficiency was further improved with the use of a higher laser fluence, and the treatment depth was improved to 3.5 mm with the use of 1,064 nm laser light at a fluence of 150 mJ/cm^2^. None of the laser-only and ultrasound-only treatments were able to remove any melanoma cells. The treatment outcome was validated with histological analyses and photoacoustic imaging. This study opens the possibility of USaLT for melanoma that is currently treated by laser therapy, but at a much lower laser fluence level, hence improving the safety potential of laser therapy.

## Impact statement

Non-invasive and agent-free therapy is always needed for melanoma. Laser therapies, widely used in melanoma treatment, require an injection of external agents and high laser power. Further, focused ultrasound therapy is another established treatment for melanoma based on hyperthermic effects to induce cell death. This is a pioneer study that uses *Ultrasound-assisted Laser Therapy*, a novel technology based on photo-mediated ultrasound therapy, a combined laser and ultrasound therapy for treating melanoma at low laser fluence per pulse requiring no external agents.

## Introduction

Melanoma is an invasive and aggressive type of cancer most frequently occurring on the skin [1, 2], as well as in the eye [3–5]. The global incidence of newly diagnosed cases of melanoma skyrocketed to 300,000 in 2020 [6]. About 90% of uveal melanoma, the most common eye cancer constituting 3–5% of all melanomas and aggressive cancer with 50% of patients dying from metastasis, is choroidal melanoma [4, 5]. Laser therapies based on thermal effect or release of reactive oxygen species have been used for treating melanoma in both skin and eye [7, 8]. These therapies are advantageous as they are minimally or non-invasive and easy to use [9].

Laser therapies used for melanoma include photothermal therapy (PTT), photo-biomodulation (PBM), transpupillary thermal therapy (TTT), and photo-dynamic therapy (PDT). PTT involves the use of photothermal agents to produce heat upon laser irradiation to treat tumor metastasis through targeted therapy [10]. PTT with a NIR laser at 240 J/cm^2^ laser fluence guided by photoacoustic imaging using hyaluronan (HA)-coated FeOOH@polypyrrole (FeOOH@PPy) nanorods as theranostics agent demonstrated anticancer activity in melanoma both *in vitro* and *in vivo* [11]. PTT at 808 nm using functionalized gold nanoparticles on melanoma cells *in vitro* was able to induce apoptosis, necroptosis, and necrosis by controlling the temperature rise through varying laser power from 0.95 W to 1.59 W for 15 min [12]. Another study on dose-dependent PTT using gold nanostars at 808 nm concluded that caspase-3-dependent apoptosis was induced at 720 J/cm^2^ laser fluence [13]. Further, a computed tomography-guided synergistic resveratrol-coated gold nanoflowers induced apoptosis and PTT at 808 nm with a laser fluence of 594 J/cm^2^ was able to kill cancer cells and showed no recurrence for 16 days *in vivo* [14]. PTT often raises safety concerns as it involves the injection of foreign agents and poses a challenge in completely eliminating the effects of hyperthermia.

PBM is the use of a low-level laser to induce modulation in physiological function without any heating to the cells and tissue [15]. A 660 nm red laser at 150 J/cm^2^ was able to inhibit cell migration and reduce VEGF production in the melanoma cells *in vitro,* and also arrest tumor progression with an increased survival rate in mice *in vivo* [16]. Another study with a red light at 635 nm was able to elevate reactive oxygen species and p53 phosphorylation in melanoma cells *in vitro* increasing apoptosis at 1280 J/cm^2^ [17]. Red light also inhibits melanoma progression and elevated CD103+ expression of dendritic cells *in vivo* at 2560 J/cm^2^. Further, laser therapy at 660 nm with a fluence of 3 J/cm^2^, and at 800 nm and 970 nm with a fluence of 6 J/cm^2^ demonstrated an increase in type I interferon both *in vitro* and *in vivo* on melanoma [18]*.* In addition, PBM was able to reduce tumor progression and elevate metabolism *in vitro*. Further, the treated area *in vivo* was surrounded by lymphocytes and dendritic cells, and mature vessels with reduced pro-angiogenic macrophages were observed.

TTT is extensively investigated for laser-based thermal therapy for choroidal melanoma. In TTT, a near-infrared diode laser (810 nm) is used to generate temperatures between 45 and 65°C through heating effect [19]. TTT affects the tumor at 0.7–2 mm depth [20]. TTT at 810 nm demonstrated complete tumor resorption in 29% of the patients with recurrent and residual choroidal melanoma [21]. In addition, TTT also resulted in complications in 44% of patients at 45-month follow-up after three treatment sessions [22]. Further, the tumor recurrence rate in patients was 11% and 15% at 5 and 10 years after TTT, and it was found to be correlated to the high-risk tumor features [23].

PDT is another laser-based therapy used for both skin and choroidal melanoma that is not based on thermal effect. PDT uses photosensitizers to release reactive oxygen species when exposed to visible light [24]. Photosensitizers are substances that can be selectively absorbed by metabolically active tissue like melanoma [25]. The reactive oxygen species induce apoptosis selectively in melanoma. PDT at 673 nm with 10 J/cm^2^ using an antibody-metallated phthalocyanine-polyethylene glycol-gold nanoparticle drug conjugate as a photosensitizer demonstrated increased cytotoxic and late apoptotic cell deaths in melanoma cells *in vitro* [26]. PDT at 450 nm using flavin mononucleotide demonstrated apoptosis on melanoma cells at 5 J/cm^2^ laser fluence *in vitro* and melanoma xenograft regression at 20 J/cm^2^ *in vivo* [27].

Several clinical studies evaluated the potential of PDT for treating choroidal melanoma. A clinical study of muti-dose PDT at 100 J/cm^2^ laser fluence for 166s per spot using verteporfin for choroidal melanoma demonstrated tumor reduction in 83% of patients after multiple sessions [28]. Another clinical study involving PDT with verteporfin for choroidal melanoma demonstrated 61.6% complications after years [24]. Another clinical study of three sessions PDT at 50 J/cm^2^ with a double duration treatment of 83 s each for choroidal melanoma with verteporfin concluded that 20% of patients failed 5 months post-treatment requiring radiotherapy [29]. The follow-up for this clinical study indicated 62% tumor regression in the 29th month following three sessions of PDT [30]. Another study used fluorinated-functionalized polysaccharide-based nanocomplexes mediated PDT for choroidal melanoma elicited photocytotoxicity through elevation of reactive singlet oxygen at 650 nm for a laser fluence of 100 J/cm^2^ for 8 min *in vivo* [31]. Even though PDT is widely used and explored for skin melanoma and choroidal melanoma, the systemic injection of the photosensitizer during PDT makes the skin photosensitive to light after the treatment and requires laser fluence over 50 J/cm^2^ for choroidal melanoma. Further, PDT is also limited to a strict treatment window due to the circulation time of the photosensitizer and a challenge with complete elimination from circulation.

The selectivity of laser therapy to treating melanoma can be achieved through selective photo-thermolysis that involves specific targeting of pigmented elements within the target by exposing them to light wavelength corresponding to the peaks of their optical absorption spectrum [32, 33]. Melanin is a pigment present in the melanosome of skin and eye [34]. By using a laser with a wavelength and pulse duration appropriate to the melanin characteristics, pigment destruction of melanin can be induced through selective photo-thermolysis [35]. The shorter wavelength lasers – pulsed dye laser and ruby laser can only treat superficial melanosomes, whereas the ruby laser is minimally absorbed by hemoglobin offering a greater selectivity for melanin [36, 37]. For the melanin pigment, the theoretical optimal optical wavelength ranges from 500 to 600 nm [38]. However, the 500–600 nm range has a limited penetrating depth in the order of 1.5 mm due to high optical absorption [38]. The near-infrared (NIR) laser has also been used in thermal therapy for melanoma, but it often involves localized injection of indocyanine green dye to improve heat generation [39–41]. Further, at longer wavelengths such as 1,064 nm, the laser light can penetrate relatively deeper tissues but at a comparatively higher laser fluence [42–44].

Ultrasound therapies are also widely explored and used for tumor treatment. Focused ultrasound (FUS) technology using a high-intensity focused transducer (HIFU) is increasingly used to non-invasively treat solid tumors [45], including melanoma [46]. Tumor cells are killed in HIFU ablation through thermal effect based on acoustic absorption, or mechanical effect based on cavitation [47]. In the event of bubble collapse, localized tissue damage is induced due to the combined effect of applied ultrasound pressure from FUS, shear stress, and high temperature due to cavitation. However, FUS therapy is not as highly selective as laser therapies because there often exists a sharp optical absorption contrast between different types of tissues.

We have recently developed a combined ultrasound and nanosecond pulsed laser technology, termed photo-mediated ultrasound therapy (PUT), to enhance cavitation activity and demonstrated its high efficiency and excellent selectivity [48–51] to remove micro-vessels. With spatiotemporally synchronized laser pulses and ultrasound bursts, the laser energy and ultrasound amplitude used in PUT are significantly less than the traditional laser-only and ultrasound-only-based technologies.

Based on the same principle, in this study, we presented an ultrasound-assisted laser therapy (USaLT) for melanoma cell removal. Different from previous therapies, such as PTT, PBM, TTT, and PDT, where continuous wave (CW) laser was used, USaLT is based on nanosecond laser pulses with a relatively low fluence per pulse. The possible underlying mechanism of USaLT is to promote cavitation and selectively destroy melanoma cells through mechanical force, rather than thermal effect, by using synchronously applied nanosecond laser pulses and ultrasound bursts. By synergistically applying ultrasound bursts, cavitation activity induced by optical absorbers like melanin during laser therapy can be enhanced [52]. USaLT is advantageous as it is highly precise resulting in high selectivity on the target by exploiting the high optical contrast between endogenous agents like melanin and other tissue in the visible to near-infrared region [52–54]. Further, USaLT is a non-invasive and agent-free technique and is designed to enhance the mechanical effect while suppressing the thermal effects by selecting optimal laser and ultrasound parameters [55].

In this study, we explored the application of USaLT for melanoma cell removal *ex vivo* by using melanin present in melanosomes as an optical absorber for targeted tumor removal. With a novel *ex vivo* melanoma model, we demonstrated that, with the assistance of FUS, laser therapy can destroy melanoma cells selectively with a much lower fluence in comparison with traditional laser therapy, and achieve a treatment depth of at least 3 mm, potentially improving the safety feature of laser therapy.

## Materials and methods

### *Ex vivo* USaLT system setup

**FIGURE 1:**
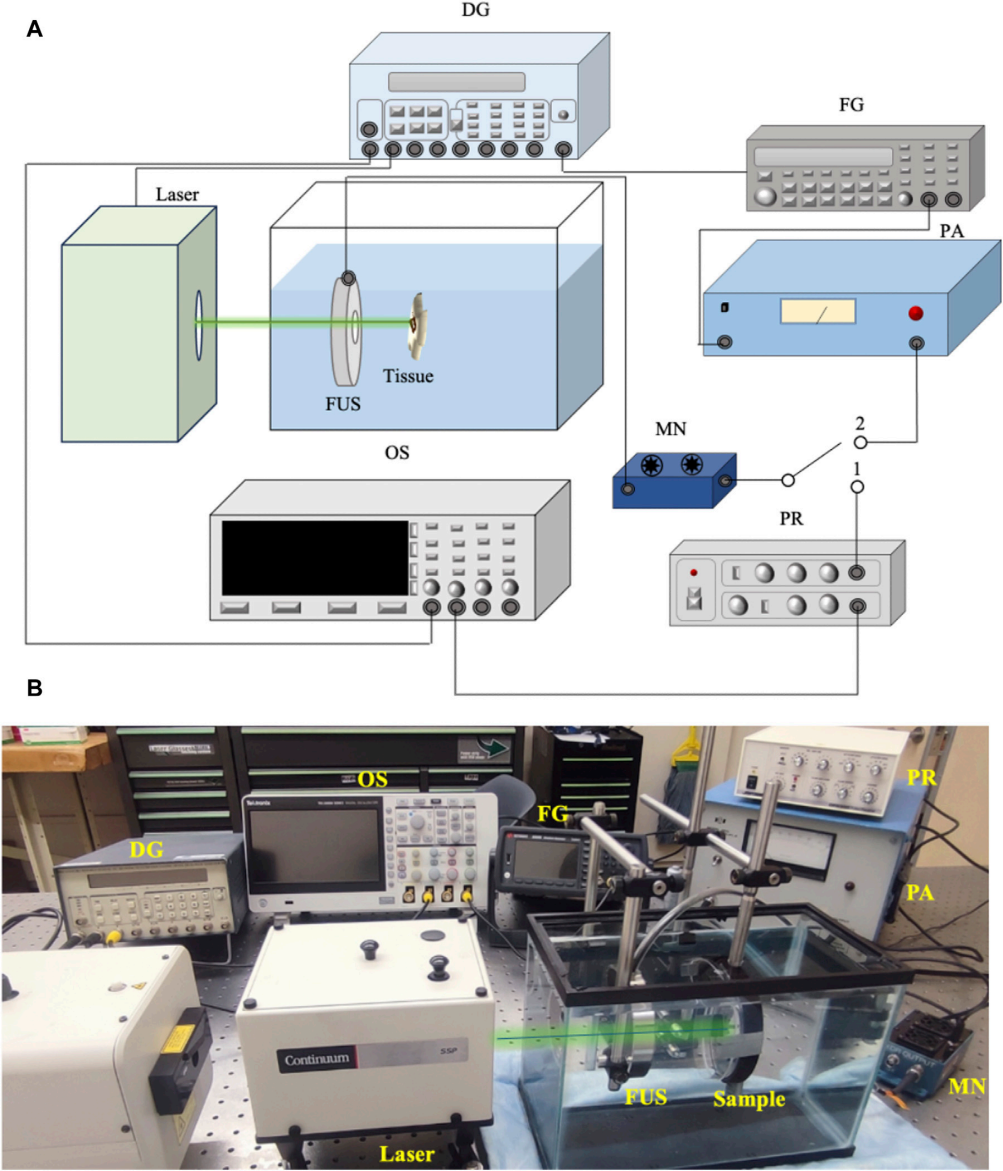
**(A)** Schematic of the system setup for USaLT. Switch position 1 is for alignment. Switch position 2 is for treatment. **(B)** Photograph of the USaLT experimental setup. DG, Delay Generator; OS, Oscilloscope; FG, Function Generator; PR, Pulse Receiver; FUS, Focused ultrasound; PA, Power Amplifier; MN, Matching Network.

Figure 1A shows the schematic of the USaLT experimental setup and Figure 1B shows a photograph of the experimental setup. A 500 kHz FUS transducer (H107, Sonic Concepts, Bothell, WA, United States) and an Nd:YAG laser (Surelite SLI-30, Continuum, Santa Clara, CA, United States) were used for USaLT. For the *ex vivo* experiment in a melanoma tumor model on the chicken breast tissue, the laser system, FUS system, and oscilloscope were triggered by a pulse delay generator (Model DG355, Stanford Research Systems, Sunnyvale, CA, United States) at a 30 Hz repetition rate.

Before each experiment, the 500 kHz FUS transducer was first spatially aligned with the laser beam which had a pulse repetition rate of 30 Hz and a pulse duration of 5 ns. During the alignment process, switch 1 position was used, and the photoacoustic (PA) signal generated upon illuminating the target with the laser beam was detected by the FUS transducer. The signal acquisition was then performed using a digital oscilloscope (DPO 3034, Tektronix Inc., Beaverton, OR, United States). The FUS transducer was scanned across the region of interest and fixed at the location where the maximum PA signal was detected, indicating the overlap of the laser and the focal region of the FUS transducer. Through this process, FUS bursts and laser pulses were spatially aligned for each sample.

Switch 2 position was then used for USaLT. The ultrasound signals were supplied by a function generator (33250A, Agilent Technologies, Santa Clara, CA, United States). The signals were then amplified using a power amplifier (350L RF Power Amplifier, ENI Technology Inc., Rochester, NY, United States) by 50 dB and fed into the FUS transducer via an impedance-matching circuit (Impedance Matching Network H107, Sonic Concepts, Bothell, WA, United States). The FUS transducer focal region was focused on the target. The focal length, focal width, and radius of curvature of the FUS transducer are 21.02, 3.02, and 63.2 mm respectively. The ultrasound duty cycle used in this study was 10% to reduce the heat generation with 1,650 cycles at a 30 Hz pulse repetition rate.

**FIGURE 2:**
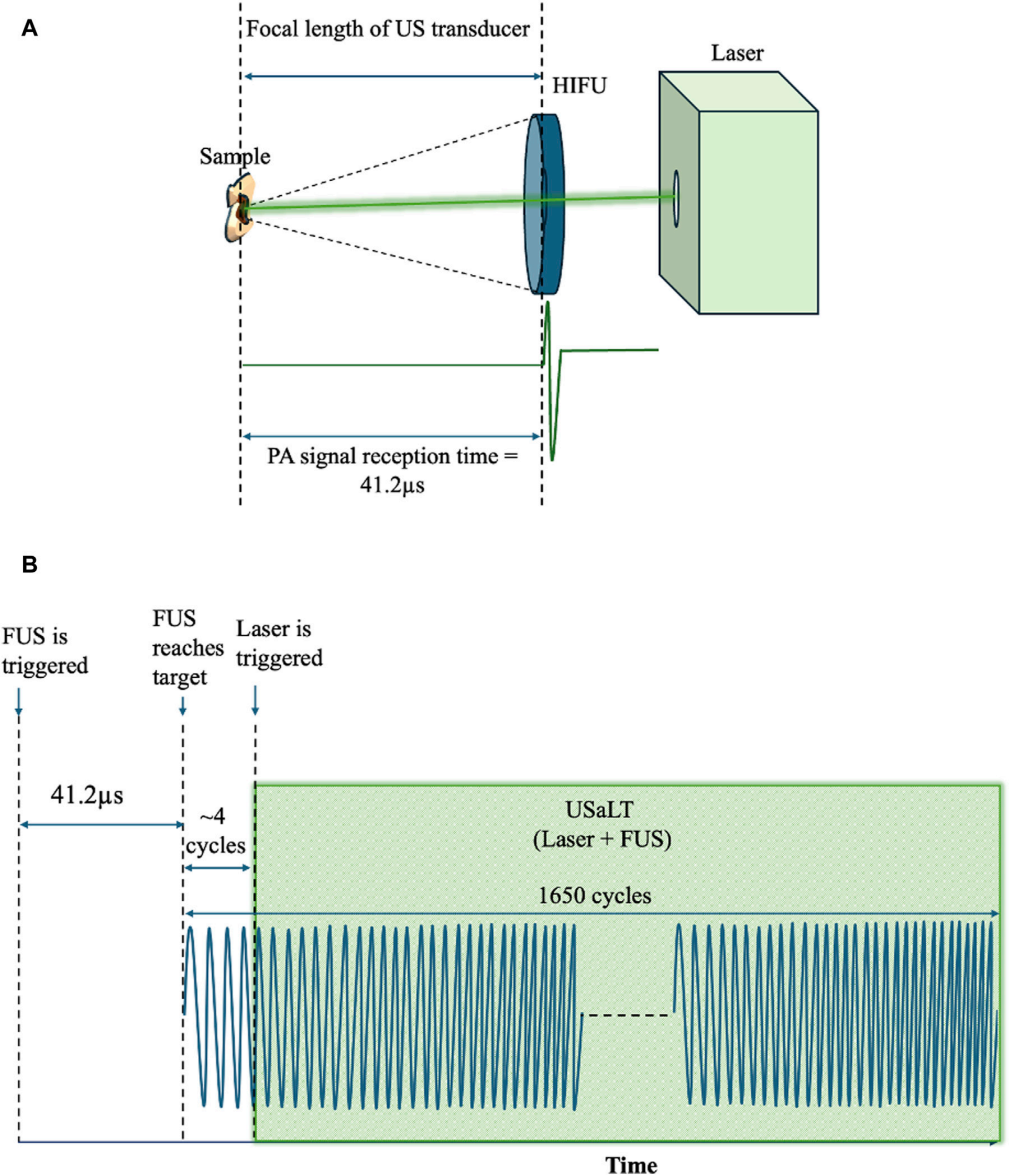
**(A)** Spatiotemporal alignment of laser and ultrasound on the sample. **(B)** FUS and laser pulses triggered during USaLT treatment using the delay generator.

For USaLT, laser pulses and ultrasound bursts were synchronously applied by controlling the laser intensity at the surface to maintain the desired laser fluence and using the same ultrasound parameters used for ultrasound-only treatment. By following a previous study [55], the timing sequence of the laser pulses and FUS bursts were precisely controlled by a pulse delay generator as shown in Figure 1. A delay time after each FUS burst was set for triggering each laser pulse such that the FUS burst can propagate to the target before a laser pulse was fired. This delay time was set based on the ultrasound traveling time from the FUS transducer to the target. To measure this traveling time, the FUS transducer was initially used in receiving mode, while a laser pulse was used to generate PA signal from the target (Figure 2A). The measured delay between the laser trigger and the FUS detected PA signal, which is the ultrasound traveling time between the FUS transducer and the target, was precisely calculated and applied to the laser system via the pulse delay generator. As a result of the synchronization, the laser pulse was anticipated to irradiate the target when the negative phase of the FUS burst reached the target, as shown Figure 2B. The diameter of the laser beam used for the laser-only and USaLT was 6 mm for *ex vivo* treatment. The laser power was monitored using an optical power meter before each treatment.

**FIGURE 3:**
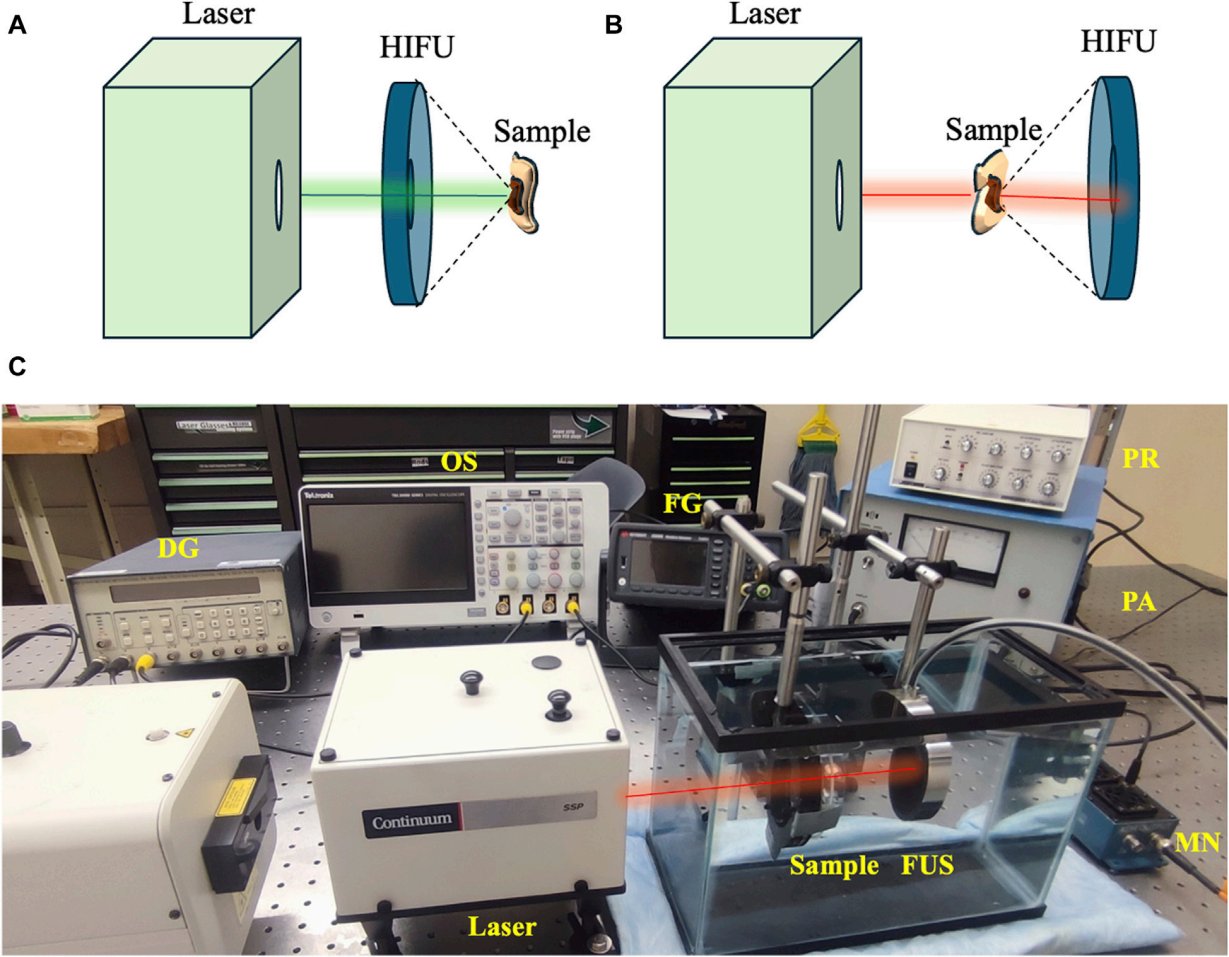
Variations in treatment setup for using **(A)** 532 nm and **(B)** 1064 nm. **(C)** USaLT photograph for using 1,064 nm. USaLT photograph for using 532 nm has been shown in Figure 1.

At 532 nm optical wavelength, the laser fluences used for *ex vivo* experiments were 20, 28, and 42 mJ/cm^2^, while 150 mJ/cm^2^ laser fluence was used for 1,064 nm optical wavelength. These parameters were selected based on a titration process in separate *in vitro/ex vivo* experiments and the availability of the laser sources. Ultrasound peak-negative pressure (PNP) of 2 MPa was used separately for laser-only and ultrasound-only treatment groups and in combination for USaLT groups (n = 5). The treatment time for each sample was 5 min. The variations of system setups for using 532nm and 1,064 nm are shown in Figures 3A, B. Figure 3C shows the photograph of the setup for using 1,064 nm.

### Cell culture

Murine melanoma cells B16F10 (ATCC^®^ CRL-6475™) were used. The cells were cultured in complete Dulbecco’s Modified Eagle’s Medium (DMEM) (ATCC^®^ 30-2002™) supplemented with 10% (v/v) Fetal Bovine Serum (FBS) (ATCC^®^ 30-2020™) and 2% (v/v) penicillin-streptomycin solution (ATCC^®^ 30-2300™) and maintained at 37°C in a humidified incubator (Nunc™, Thermo Scientific, Waltham, MA, United States) with 5% CO_2_ in a 75 cm^2^ flask. The cells were split into a 1:10 ratio once 80% confluence was reached.

### *Ex vivo* chicken breast melanoma tumor model

The melanoma cells were grown on chicken breast tissue. The chicken breast was cleaned and cut into pieces 3.5 mm thick. The cells were then injected into the tissue at a concentration of 2 × 10^6^ cells/mL. The cell numbers used for melanoma preparation into the tissue were the minimum concentration needed to demonstrate any visible cell growth, analyzed upon optimization through injecting various concentrations. The cells were injected in one shot using a 1 mL syringe with the needle bevel completely inserted facing upwards about 1.75 mm into the tissue at 45°. The tissue injected with the cells was then incubated in the refrigerator for 7 days. The tissue samples were then washed with phenol-red-free DMEM (31053028, Gibco™) before treatment. The samples were placed in a complete cell culture medium for hydration after injection. Further to avoid tissue dehydration and degradation of tissue fat at higher temperatures, samples were placed in a refrigerator during adhesion and growth. The tissue samples were placed in 4% formaldehyde in the medium after treatment until histology procedure.

### Photoacoustic imaging for evaluation of treatment effect *ex vivo*

**FIGURE 4:**
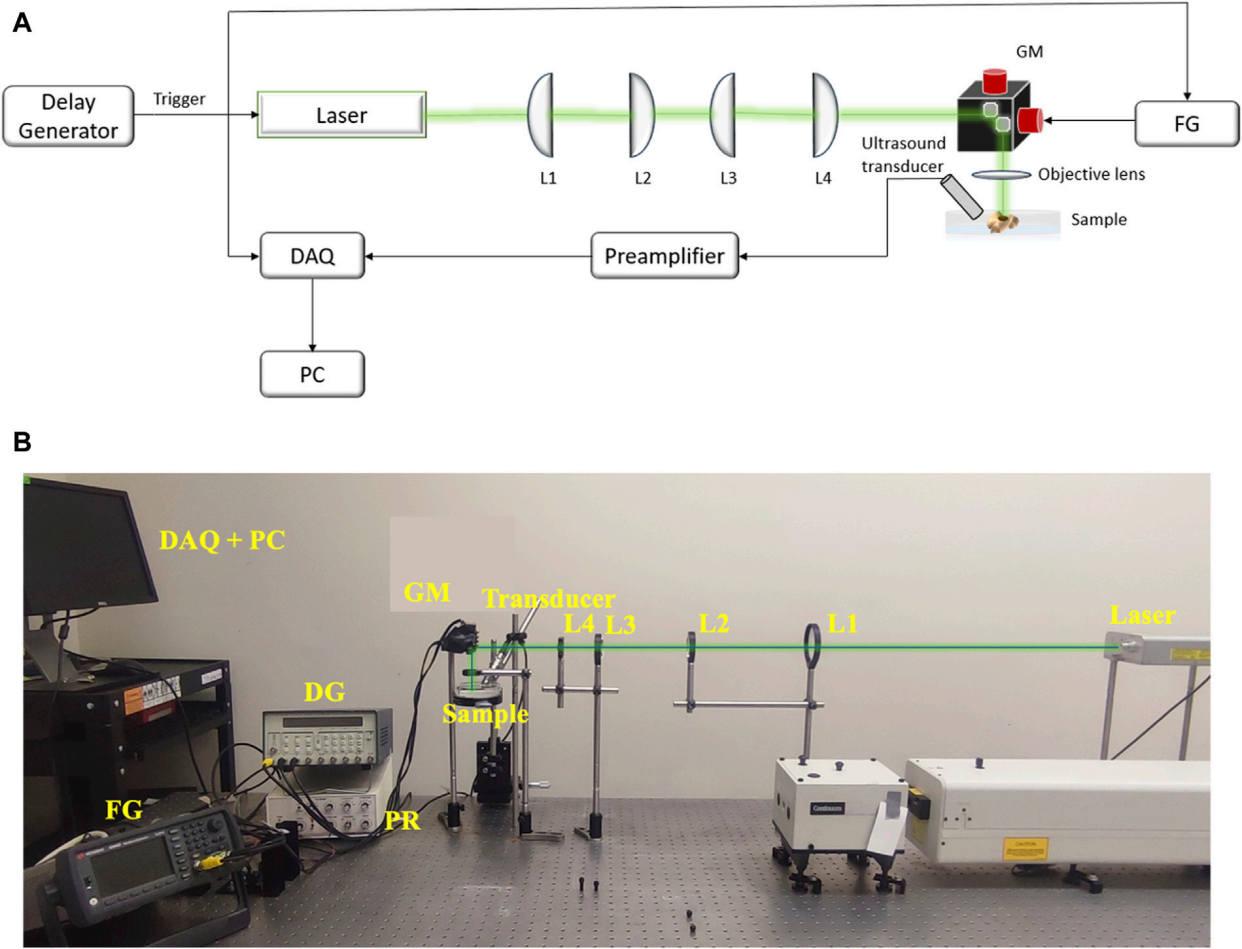
**(A)** The schematic and **(B)** photograph of system setup for PA imaging. FG: Function Generator, DAQ: Data Acquisition Card, PC: Computer, GM: Galvanic mirror, L1 (f = 150 mm), L2 (f = 100 mm), L3 (f = 75 mm), L4 (f = 25.4 mm): Lenses.

An optical-resolution PA microscopy system (Figure 4) was used to evaluate the treatment outcome on the melanoma cells *ex vivo*. A Q-switched Nd:YAG (532 nm, SPOT-10-100-532, Elforlight, Daventry, UK) laser with a pulse width of 2 ns and pulse repetition rate of 20 kHz pulse repetition was used to produce PA signals. The laser light passed through a series of planoconvex lenses – L1 (f = 150 mm, LA1002-A, Thorlabs, New Jersey, United States), L2 (f = 100 mm, LA1050-A, Thorlabs, New Jersey, United States), L3 (f = 75 mm, LA1145-A, Thorlabs, New Jersey, United States) and L4 (f = 25.4 mm, LA1951-A, Thorlabs, New Jersey, United States). A 2.25 MHz center frequency flat ultrasound transducer (V323-SU, Olympus NDT, Waltham, MA) captured the PA signals generated from the sample. The signal detected by the transducer was delivered to the pre-amplifier (DPR300, Olympus-NDT, Waltham, MA), and finally collected by a personal computer through an A/D data acquisition card (Octopus CS8289: OCT-828-009, GaGe, Lockport, IL) at a sampling rate of 10 MHz. Each sample was placed in degassed water during imaging process, and the PA images of the region of interest were acquired through raster scanning of the laser beam using a galvanic mirror (GVS202, Thorlabs, New Jersey, United States) and focused on the sample through an objective lens (f = 30 mm, LB1757-A, Thorlabs, New Jersey, United States). The laser trigger was used to synchronize the acquisition and the scanning system. A two-dimensional image was reconstructed using the acquired signals from 2D scanning. The images of the *ex vivo* melanoma tumor in each sample were acquired before and after the treatment. The PA intensity was calculated as average over the segmented tumor area as the treatment area of USaLT covered the entire tumor region. The relative change in PA intensity before and after the treatment was used for evaluating the treatment effect. All the quantitative signal analyses were performed on the raw data before any post-processing.

### Sample preparation for staining

The samples were fixed in 4% formaldehyde immediately after the treatment. The 4% formaldehyde was prepared by diluting 16% formaldehyde (Sigma Aldrich, Cat. No. P6148, Lot No. MKCD5277) aqueous stock solution. The histology procedure for paraffin embedding was performed using a Pelco BioWave^®^ Pro 36,500 Laboratory Microwave System with a Pelco^®^ Steady Temp™ Pro Thermo Cube (Ted Pella, Inc.). The samples were first dehydrated with increasing concentration of alcohol at 40°C, 5 min each: 50% ethanol, 70% ethanol, 95% ethanol, 1:1 95% EtOH/Isopropanol, and finally at 100% isopropanol before infiltrating with 1:1 isopropanol/paraffin for 5 min at 70°C. Then, samples were embedded in paraffin (Leica Paraplast Plus^®^, Cat. No. 39602004, Lot No. 2207122) thrice at 70°C with vacuum for 5 min, 10 min, and 5 min respectively. After 24 h, samples were sectioned using a rotary microtome; sections were ranging between 6 μm and 8 µm thickness. Microscope slides were manually coated with a gelatin solution, in which 1 g of gelatin powder, type A (Electron microscopy Sciences [EMS], Cat. No. 16584, Lot No. 150226) was dissolved in 1L of hot distilled water and then mixed with 0.1 g of chromium (III) potassium sulfate (Sigma-Aldrich, Cat. No. 243361, Lot No. MKBV2677V) once it cooled down. These slides were kept at 4°C until use. The sample sections were then mounted on these coated microscope slides; 3 to 4 sections were mounted per slide.

**FIGURE 5:**
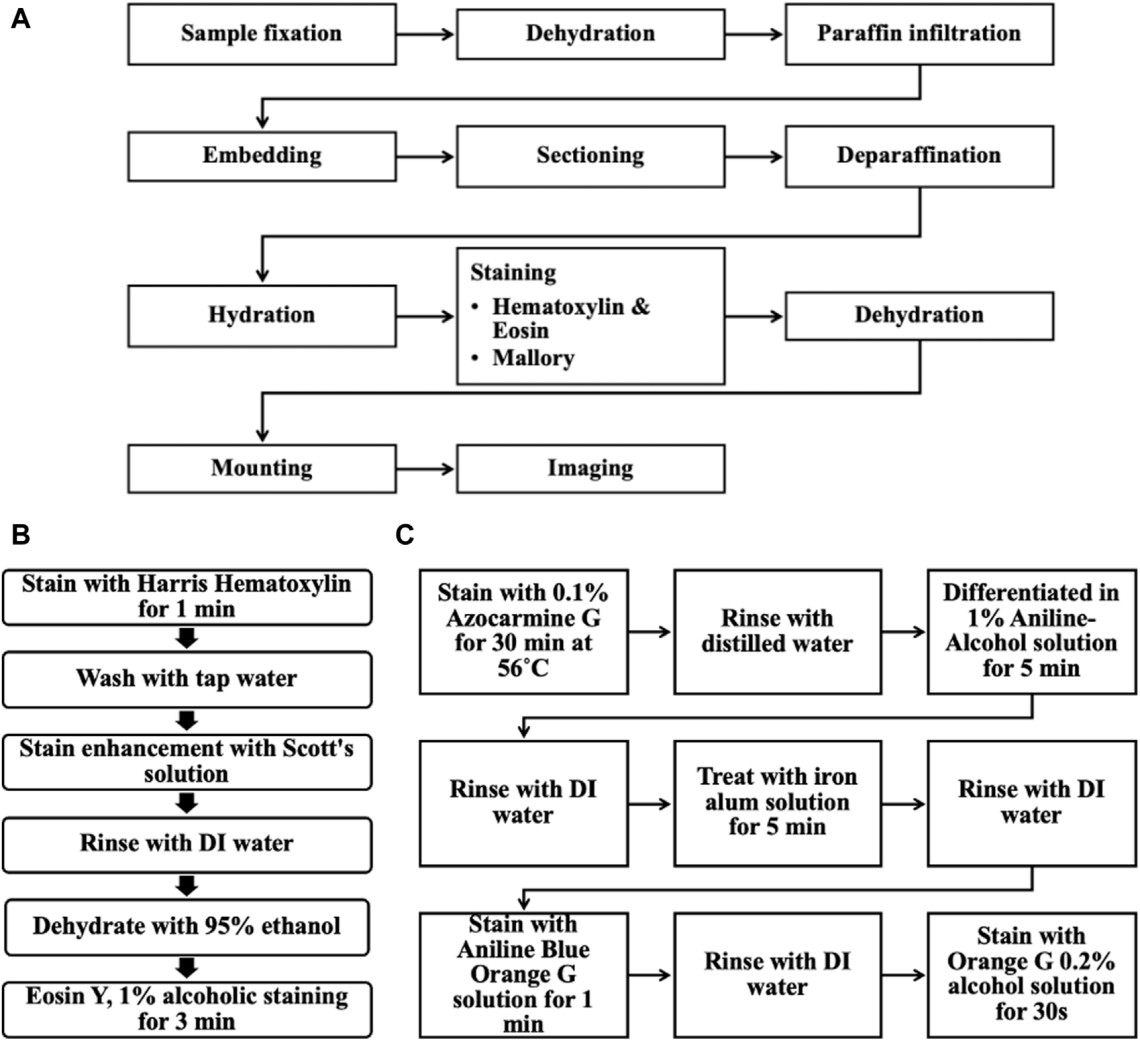
**(A)** Overall workflow of the histological procedure for the *ex vivo* chicken breast melanoma tumor model. **(B)** Steps for Hematoxylin & Eosin staining. **(C)** Steps for Mallory stain.

Sample sections were deparaffinated, hydrated, stained, and dehydrated using a HistoPro^®^ 414 Linear Stainer for paraffin and frozen sections (RUSHABH Instruments, LLC.). The samples were deparaffinated using Histo-clear^®^ II (National Diagnostics, Cat. No. HS-202, Lot No. 11-19-38) for 2 min in the first step, followed by 25 dips in Histo-clear^®^ II two times. The sample sections were then hydrated through 25 dips in the following solutions: 100% ethanol, 100% ethanol, 95% ethanol, 70% ethanol, and in running tap water for 1 min. Figures 5A–C shows the overall workflow for the histology procedure.

### Hematoxylin and eosin staining

Sample sections were stained in Harris Hematoxylin (EMS, Cat. No. 26754, Lot No. 211111-04) for 1 min and washed in running tap water for 1 min. This hematoxylin staining was enhanced using Scott’s solution next, dipping 25 times; this solution was prepared by adding 2.0 g of sodium bicarbonate (Sigma Life Science, Cat. No. S6014, Lot No. 127K0680) and 20.0 g of magnesium sulfate heptahydrate (Fisher Chemical, Cat. No. M63-500, Lot No. 185221) to 1 L of distilled water. The sample sections were rinsed in DI water, and 95% ethanol was dipped 25 times each and stained with Eosin Y, 1% alcoholic (EMS, Cat. No. 26762-01, Lot No. 211111-05) for 3 min. The sample sections were then initially dehydrated by dipping in the following solution 25 times: 95% ethanol, and 95% ethanol.

### Mallory-Heidenhain Azan-Gomori’s modification staining

Alternating microscope slides from each sample were selected for Mallory-Heidenhain Azan-Gomori’s Modification staining. The sample sections were then stained in 0.1% Azocarmine G (EMS, Cat. No. 26450-01, Lot No. 230725-21) for 30 min at 56°C (staining glass jar was placed inside an oven set at this temperature). Then, sample sections were rinsed with distilled water (25 dips), differentiated in Aniline-Alcohol 1% solution (EMS, Cat. No. 26450-02, Lot No. 230725-22) for 5 min, rinsed with distilled water (25 dips) and transferred to an iron alum solution (Ferric Ammonium Sulfate, 5% aqueous; EMS, Cat. No. 26450-03, Lot No. 230724-23) for 5 min, rinsed again (25 dips) and stained with Aniline Blue-Orange G solution (EMS, Cat. No. 26450-04, Lot No. 230725-24) for 1 min. The sample sections were briefly rinsed with distilled water (5 dips) and blotted carefully to then dehydrated completely in 100% ethanol and stained with Orange G 0.2% alcohol solution (EMS, Cat. No. 26450-05, Lot No. 230724-25) for 30 s. This staining protocol was optimized using a mouse pancreas before running these samples (Supplementary Figure S1).

### Dehydration and imaging

The sample sections were completely dehydrated by dipping in the following solution 25 times: 100% ethanol, 100% ethanol, Histo-clear^®^ II, and Histo-clear^®^ II. Finally, the samples were placed in Histo-clear^®^ II before mounting and sealed with cover glass using Permount^®^ mounting medium. These sample sections were imaged using a Nikon Eclipse LV100D-U compound bright field upright microscope and images were acquired with Q Capture Pro Version 6.0.0.412 using Q Imaging MicroPublisher 5.0 RTV camera with a pixel resolution of 2560 × 1920 using either a Plan Flour 10X/0.30NA air objective or Plan Flour 20X/0.45NA air objective.

## Results

### Validation of *ex vivo* tumor model

**FIGURE 6:**
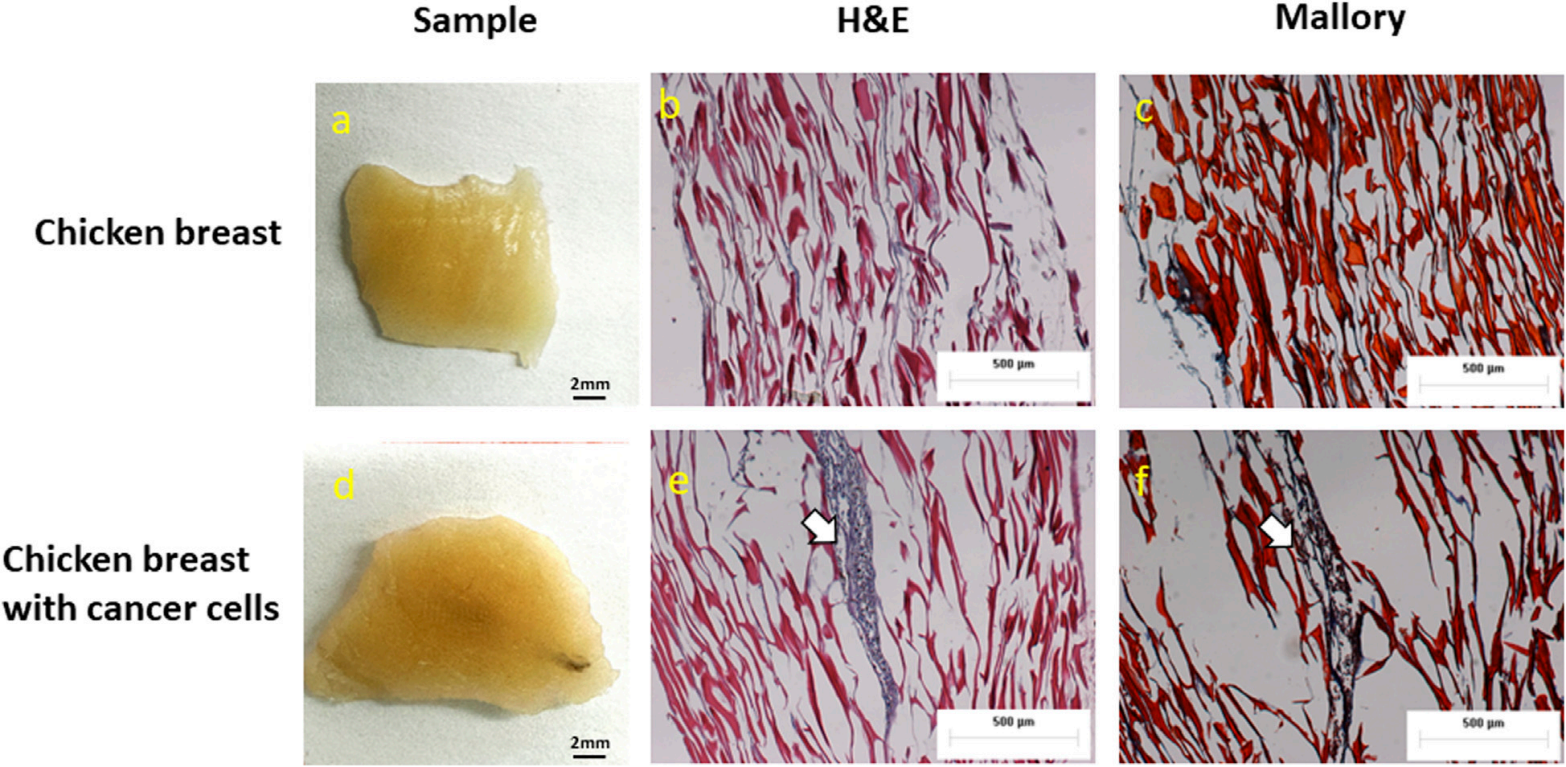
Photographs, H&E, and Mallory staining of **(A–C)** Chicken breast only and **(D–F)** *ex vivo* model of chicken breast containing melanoma cells respectively. White arrows indicate the melanoma cancer cells.

**FIGURE 7:**
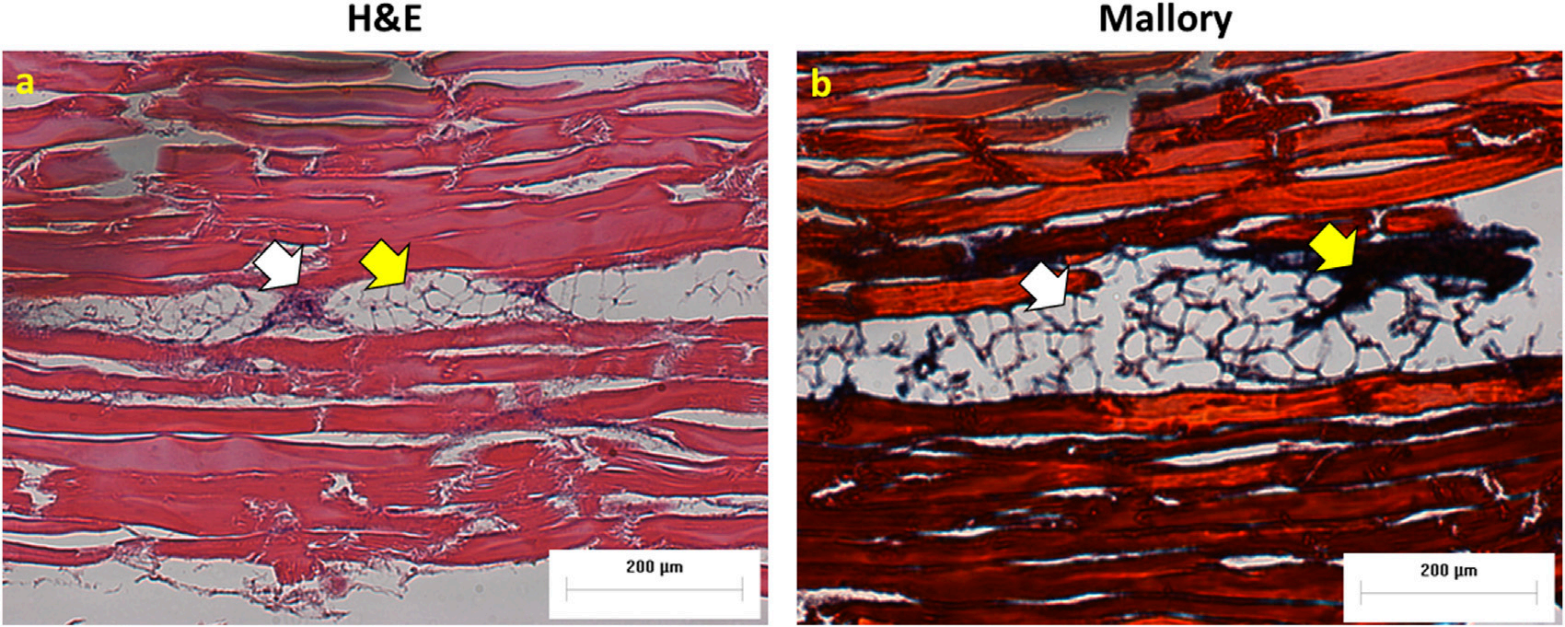
Melanoma cells attached to adipose tissue **(A)** H&E and **(B)** Mallory staining. White arrows show cancer cells and yellow arrows indicate adipose tissue.

The Hematoxylin and Eosin staining (H&E), as well as the Mallory staining, was used to confirm and validate the *ex vivo* tumor model after 7 days of melanoma cell incubation in chicken breast tissue (Figure 6). Both H&E and Mallory staining showed a group of melanoma cells holding onto the chicken tissue and forming a network. In the H&E staining, the melanoma cells were dark blue clusters, while with Mallory staining, the cells were present as a dark black cluster. This was validated by comparing them with the chicken breast with no tumor cells. Further, the melanoma cells were also found holding on to the adipose tissue when present. The adipose tissue was indicated by a patterned network using a yellow arrow (Figure 7). These results demonstrated that melanoma cells can successfully grow in *ex vivo* chicken breast tissues with our established protocol in this study.

### USaLT-induced tumor removal on *ex vivo* melanoma model with 532 nm optical wavelength

**FIGURE 8:**
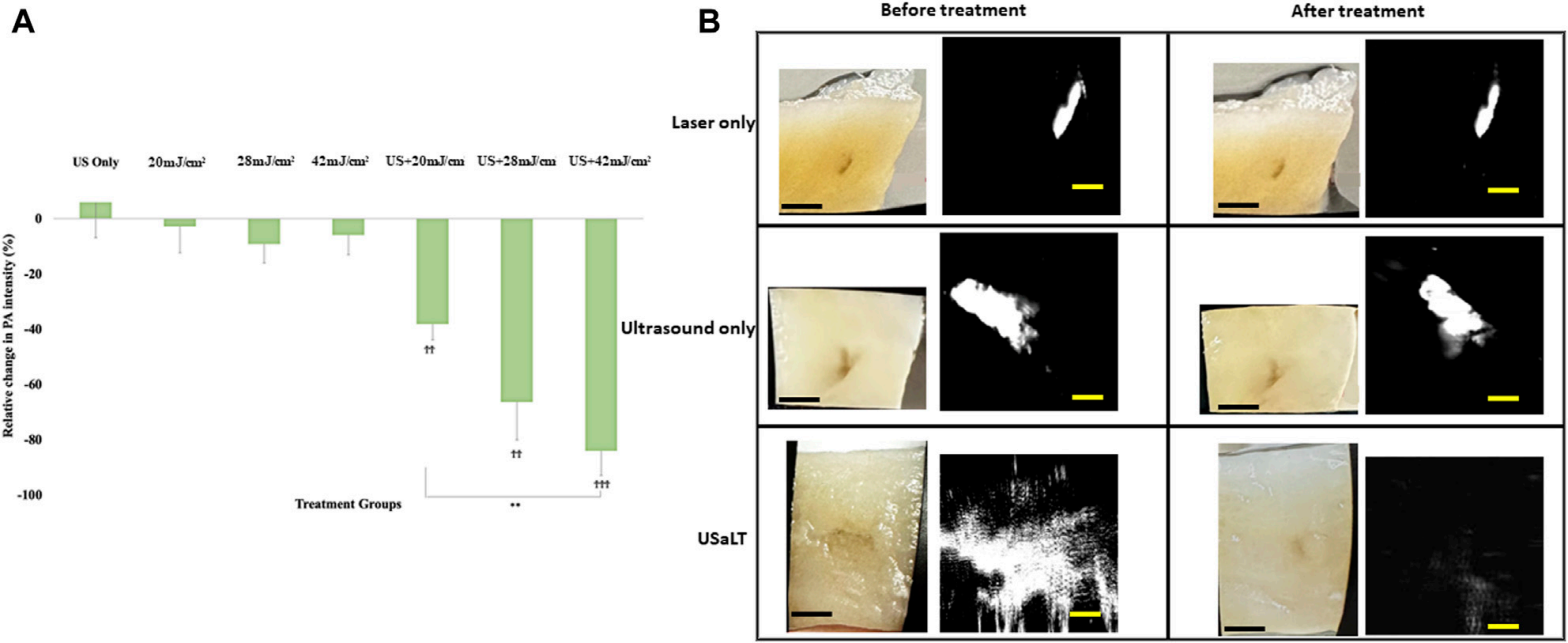
**(A)** Chicken breast photographs and photoacoustic (PA) images. Black scale bar = 2 mm; yellow scale bar = 500 µm. **(B)** Relative change in PA signal intensity for all treatment groups. “ϮϮ” and “ϮϮϮ” indicate statistical significance of p < 0.01 and p < 0.001 between control and treatment groups, respectively. “**” indicates statistical significance of p < 0.01 between treatment groups respectively. n = 5. Laser only: 42 mJ/cm^2^ light fluence at 532 nm wavelength; ultrasound only: 2 MPa PNP ultrasound pressure; USaLT: 42 mJ/cm^2^ light fluence at 532 nm wavelength and 2 MPa PNP ultrasound pressure.

**FIGURE 9:**
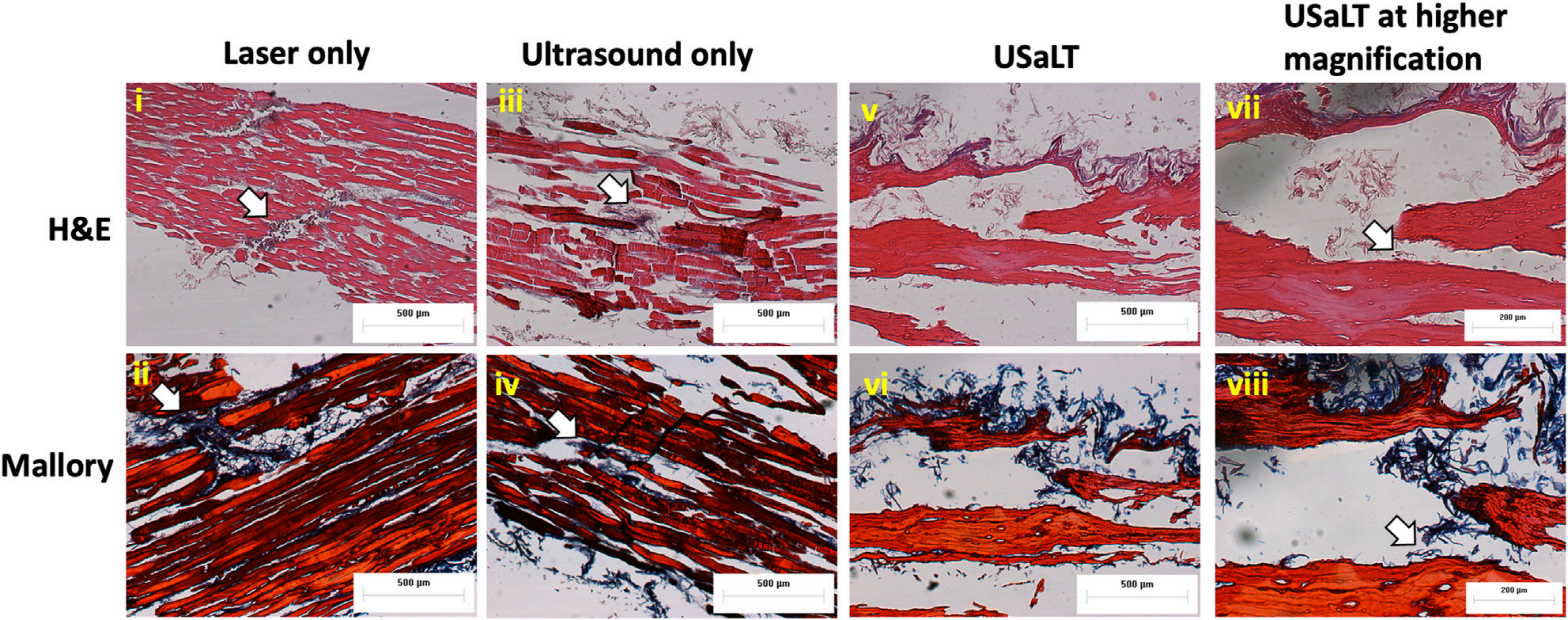
H&E and Mallory staining of (i-ii) laser only, (iii-iv) ultrasound only, and (v-viii) USaLT treatment groups. White arrows indicate the melanoma cells. Laser only: 42 mJ/cm^2^ light fluence at 532 nm wavelength; ultrasound only: 2 MPa PNP ultrasound pressure; USaLT: 42 mJ/cm^2^ light fluence at 532 nm wavelength and 2 MPa PNP ultrasound pressure.

For all the control groups with ultrasound-only and laser-only treatment, there was no significant removal of melanoma cells at the chosen treatment parameters, as validated by the acquired PA images. However, the relative change in PA intensity was significantly decreased for all USaLT groups with 2 MPa PNP and different laser fluences in comparison with before-treatment PA intensity. The average decrease in PA intensity was 38.17% at 20 mJ/cm^2^ (*p* = 0.0001, n = 5), 66.41% at 28 mJ/cm^2^ (*p* = 0.0013, n = 5), and 84.03% at 42 mJ/cm^2^ (p < 0.0001, n = 5) (Figures 8A, B). Furthermore, there was a 120% increase in reduction at 42 mJ/cm^2^ (*p* = 0.0029, n = 5) when compared to 20 mJ/cm^2^ for the USaLT groups concurrently applying 2 MPa PNP ultrasound. The H&E and Mallory staining showed only a few residual melanoma cells remaining after treatment in the USaLT group (Figure 9), while the ultrasound-only and the laser-only groups had the melanoma clusters intact after the treatment, indicating, at these power levels, ultrasound-only and laser-only were unable to remove melanoma cells, while USaLT can effectively damage melanoma cells. Further, for the USaLT group, while most of the melanoma cells were removed, the surrounding chicken breast tissue structure remained intact, demonstrating the treatment is highly selective.

### Depth analysis for USaLT on *ex vivo* melanoma model

**FIGURE 10:**
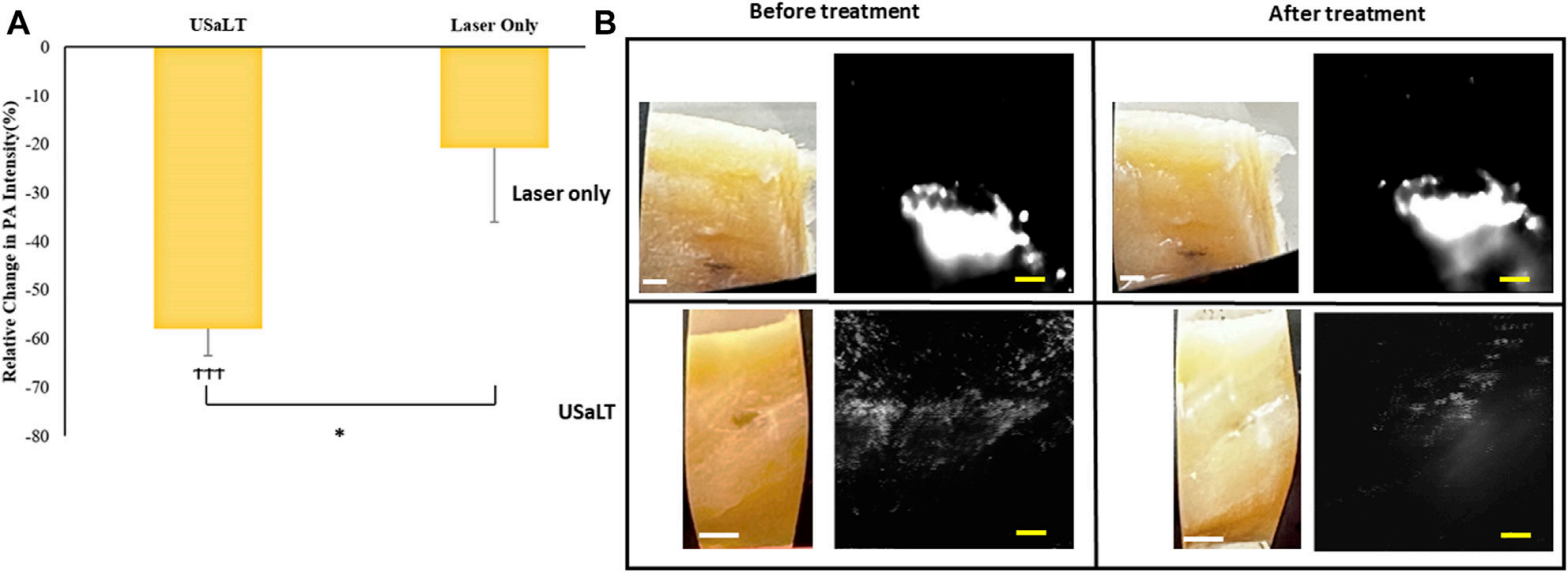
**(A)** Relative change in PA intensity (%) for all treatment groups “ϮϮϮ” indicates statistical significance with p < 0.0001 between control and treatment groups. “*” indicates p < 0.05 between treatment groups. n = 5. **(B)** Photographs and PA images of samples. Laser only: 150 mJ/cm^2^ light fluence at 1,064 nm; USaLT: 150 mJ/cm^2^ light fluence at 1,064 nm and 2 MPa PNP ultrasound pressure. White scale bar = 1 mm; yellow scale bar = 250 µm.

**FIGURE 11:**
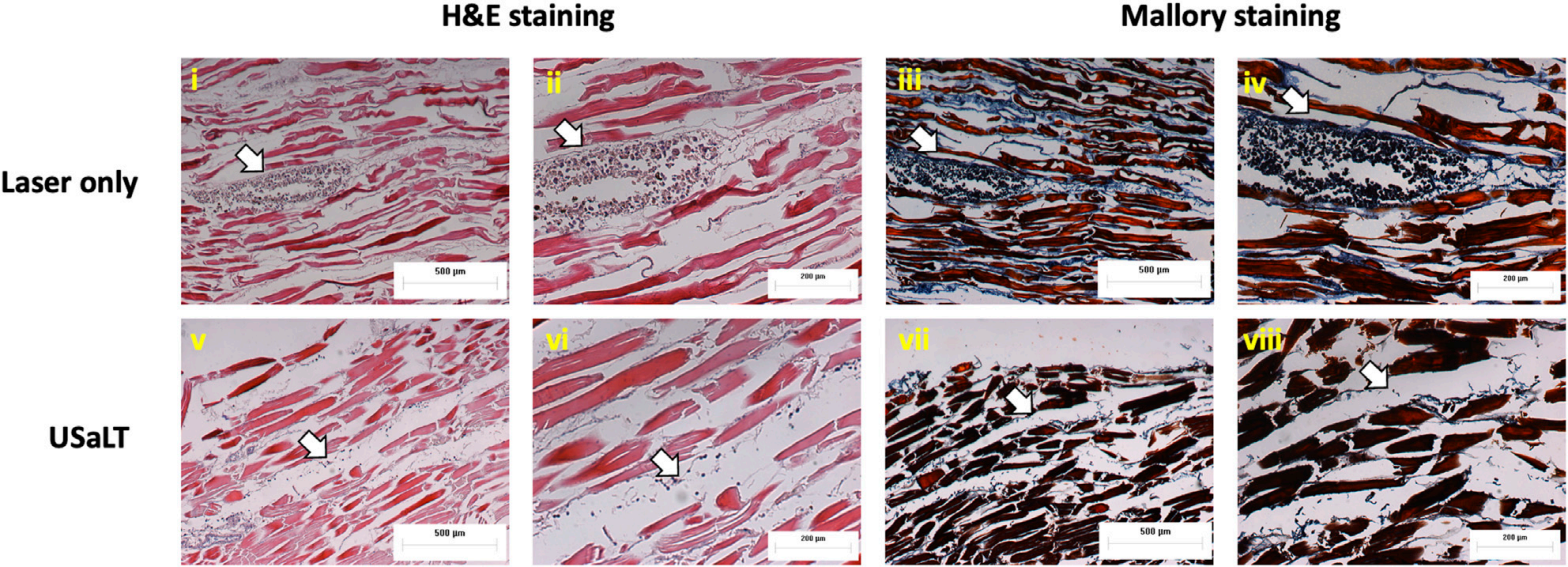
H&E and Mallory staining of melanoma treated placing at a depth of 3.5 mm. White arrows indicate melanoma cells. Laser only: 150 mJ/cm^2^ light fluence at 1,064 nm; USaLT: 150 mJ/cm^2^ light fluence at 1,064 nm and 2 MPa PNP ultrasound pressure.

The treatment results shown in Figure 8 only demonstrated a treatment depth of ∼1 mm due to the use of 532 nm. To treat deep regions, we switched to 1,064 nm optical wavelength, and the experiment was repeated. The melanoma side of sample was placed towards the laser for 532 nm laser. For the depth study, the melanoma side was placed towards the FUS transducer so that the laser beam had to pass through the entire sample of 3.5 mm before reaching the melanoma. We tested the treatment on a range of depths and found that 3.5 mm treatment depth is the maximum depth USaLT can be achieved with a laser fluence of 150 mJ/cm^2^ at the top surface (laser side) (Figures 10A, B). Melanoma cells at a depth of 3.5 mm were not significantly removed by the laser-only treatment. However, the USaLT group with 2 MPa PNP ultrasound in combination with 150 mJ/cm^2^ at 1,064 nm efficiently removed melanoma cells, resulting in a reduction of 57.96 ± 5.66% (n = 5, p < 0.001) in PA intensity (Figure 10A). The H&E and Mallory staining validated the tumor removal during USaLT with only residual melanoma cells and tumor network post-treatment (Figure 11). However, the laser-only treatment group still had the tumor structure present in both staining. Again, the chicken breast tissue structure was intact, demonstrating the treatment is highly selective.

## Discussion

We demonstrated that USaLT can selectively remove melanoma cells. At 532 nm, USaLT can be effective at as little as 20 mJ/cm^2^ laser fluence per pulse, and the ablation efficiency increases as the laser fluence increases. However, due to high attenuation, the treatment depth is only limited to ∼1 mm. With the 1,064 nm laser wavelength, where the optical attenuation of tissue is much weaker, USaLT was able to successfully remove the melanoma cells at a depth of about 3.5 mm with a laser surface fluence of about 150 mJ/cm^2^ per pulse. During the treatment depth study, the laser beam needed to pass through a ∼3 mm thickness of normal tissue before it reached the melanoma cells. The purpose of this study was to examine whether the remaining laser energy would be sufficient for USaLT after the laser beam passed through a layer of tissue. This study did not explore the potential of USaLT treatment with respect to tumor thickness. In a real situation, the melanoma may be removed layer by layer during USaLT. In this case, laser beam will always and only need to pass through normal tissue after the top layer melanoma is selectively removed.

TTT, as a currently available treatment for choroidal melanoma, can efficiently treat at a depth of about 0.7–2 mm when using external agents and laser fluences >100 J/cm^2^ [20, 22]. However, TTT causes complications such as superficial petechial and vitreous hemorrhages, macular edema, exudative and rhegmatogenous retinal detachment, retinal vascular occlusion and traction, optic disc atrophy, maculopathy, and extraocular tumor extension [21–23]. In addition, the treatment efficiency in TTT and PDT used for choroidal melanoma are also dependent on the dose of external agents raising safety concerns [13, 28]. In comparison, USaLT can reach 3.5 mm with great selectivity and is free of any external agents. While thermal-based laser therapy destroys all the cells in the heated region, USaLT selectively removes melanoma cells, and the adjacent chicken breast tissue cells remain intact.

As demonstrated in previous studies related to combining laser and FUS therapy such as PUT, the potential underlying mechanism of USaLT is enhanced cavitation. Laser-induced cavitation in high optical absorptive biological tissue, such as melanoma cells, can be further driven by the applied FUS. It is difficult or “tricky” to directly compare the laser fluence between USaLT and other existing laser therapies for melanoma because USaLT utilizes nanosecond pulsed laser while other laser therapies utilize CW lasers. In the current study, we used 5 min treatment duration, which corresponds to 180 J/cm^2^ to 1350 J/cm^2^ total laser fluence and is in a similar range with fluence used in the existing therapy. However, there is no hyperthermia effect in USaLT because a nanosecond laser is used. With great selectivity and without hyperthermia effect, USaLT can potentially offer safe and precise treatment, which can be significant for treating choroidal melanoma in the eye.

From the laser therapy point of view, this approach can avoid potential unwanted damage to surrounding tissue. From the FUS therapy point of view, ablation becomes much more selective because cavitation is only limited to optical absorptive biological tissues. In the current case, the high optical contrast between melanin and the surrounding tissue was used to achieve high selectivity eliminating the need for injection of any external agents for treatment or selectivity [56].

Similar to PUT, USaLT also involves a synchronous application of pulsed laser and ultrasound [55, 57] as both are dependent on the generation of cavitation bubbles through stress confinement rather than thermal effect and both use a lower laser fluence range. Therefore, USaLT eliminates the possibility of hyperthermic effects and the risk of scarring, which are potential challenges of currently available laser-based thermal therapies for melanoma. Hence, the melanoma destruction was achieved at a laser fluence of 150 mJ/cm^2^ per pulse when assisted by ultrasound. As the treatment efficiency for the thermal effect is dependent on the thermal relaxation time, stress confinement is dependent on stress relaxation time. Depending on the size of the melanosomes, the maximum treatment efficiency for thermal therapies could be achieved by using 0.25–1 µs lasers [35]. Moreover, for a 10 nm melanin granule, the stress relaxation time is 7 ps [58]. Hence, in the future, a picosecond laser will be used to facilitate photomechanical disruption for a more efficient melanoma treatment at reduced laser fluence similar to a study involving a 630 nm PDT-based *in vitro* melanoma treatment with a femtosecond laser, but without requiring any external agents [59].

We also presented a novel *ex vivo* melanoma tumor model in the current study. The *ex vivo* model used here is hypothesized to be feeding on chicken tissue in addition to the cell culture medium for growth. Further, the histological procedure showed that the cancer cells adhered to the adipose tissue and grew on it indicating that it could absorb nutrients that were already present in the tissue. In the future, the cell growth using various dead tissue extracts to get a clear insight. The motivation behind growing the cells in dead tissue is to facilitate the efficacy of cancer-based studies like drug and treatment evaluation when compared to cell-based *in vitro* studies. The nutrients present in the chicken breast tissue including intramuscular fat, collagen, and protein might have aided in the growth of cancer cells on them [60]. Since the model required for this study needed a visible tumor for evaluation, the injection zone is restricted to 1.75 mm from the surface. However, the cells were able to grow when injected deeply into the chicken breast.

All tumor samples were prepared applying the same procedure. Every tumor sample presented in this sample was distinct visually due to metastatic tumor growth, even though the same cell passage and same number of cells were injected and incubated for the same amount of time. In the presented histology results, the tumor was deeper which substantiates the fact that the black tumor spot visible in the photograph is restricted to the surface. Further, the laser beam size was 6 mm in diameter, and the FUS focal spot was 3 mm in diameter. Hence, USaLT, which is dependent on the spatial synchronized laser and FUS, had a treatment region of 3 mm. During all the experiments, the treatment area covered a 3-mm tumor region. The relative PA signal change was calculated for each sample before and after the treatment. These changes were compared between different samples to minimize the effect induced by the size difference between tumor samples.

Since this is the first study reporting the growth of cancer cells in dead tissue, two staining techniques H&E and Mallory were performed to validate tumor growth in the model. H&E is a gold standard histological staining used for cells and tissues; it highlights melanoma cells in brown color. Mallory is used extensively for fibrous tissue and chicken breast tissue is a muscle fiber; in addition, Mallory also stains melanoma cells and was used for substantiation of H&E stain as the model used in this study was novel.

The absorption coefficient of a single melanosome is 550 cm^−1^ at 532 nm and 55 cm^−1^ at 1,064 nm [61, 62]. The absorption coefficient of chicken breast tissue is 0.7 cm^−1^ at 532 nm [63, 64] and 0.01 cm^−1^ at 1,064 nm [65]. The difference in their optical absorption produces contrast in PA imaging and selectivity during treatment. As the difference in optical absorption between melanin and surrounding tissue is used for the selective targeting of melanoma during USaLT, a relatively higher difference will provide better selectivity. Also, it is pertinent to work at a wavelength that can provide high absorption for melanin when compared to blood. The relative absorption of melanin with respect to blood is maximum at 755 nm (54:1), followed by 1,064 nm (16:1) and then 532 nm (2.4:1) [66]. Hence, considering the penetration depth and optical absorption for selectivity 1,064 nm, it might be the ideal wavelength for further *in vivo* studies.

The study reported here is a preliminary study to evaluate the proof-of-concept for using PUT-based USaLT for treating melanoma. In this study, tumor removal using USaLT was confirmed with an *ex vivo* model. The motivation behind using the tumor model is that it allows us to examine the selectivity and depth of treatment of USaLT, which would be otherwise not possible with other *in vitro* studies. Since USaLT is based on PUT, it is hypothesized that the destruction of cells occurs through the mechanical cavitation effect due to the combined effect of laser and ultrasound which at a similar level independently cannot induce any cell damage. Detailed mechanism of action and safety study will need to be carried out *in vivo* in the future, particularly if USaLT is used to treat metastatic melanoma. The potential application of this technique to choroidal melanoma should be evaluated and compared with the current thermal-based laser therapies. Further, while the current study focused on cell death induced by USaLT, other interesting future studies are to examine whether USaLT can stimulate cell metabolism and reproduction, and promote tissue healing. With the improved treatment depth, USaLT can affect cells deeper in tissue than pure optical techniques.

Although the treatment depth can be improved in comparison with pure optical techniques, the most significant limitation of USaLT is still its depth of treatment. The current study demonstrated a treatment depth of up to 3.5 mm through chicken breast tissue. This treatment depth may significantly limit the adoption of USaLT other than for the treatment of choroidal melanoma, where a clear optical path exists.

In the future, a real-time PA image-guided USaLT system may be developed to provide instant feedback on the treatment, and the cavitation mechanism behind cell death should also be further investigated. In addition, in an *in vivo* animal melanoma model, the potential of USaLT to induce immune response should be evaluated.

## Conclusion

To summarize, this study is the first study to successfully demonstrate the potential of a PUT-based USaLT technique involving the synchronous application of laser and FUS to destroy melanoma. Initial investigation with 532 nm showed that USaLT could selectively remove melanoma. Further, USaLT showed the selective removal of melanoma cells at relatively low laser fluence per pulse. Also, at 1,064 nm, a higher optical wavelength, a treatment depth of 3.5 mm could be achieved with USaLT. The histological analysis substantiated that USaLT treatment removed melanoma cells while sparing the surrounding tissue. Further, the laser and ultrasound-only treatment at similar energy levels did not result in the removal of the melanoma. This study demonstrated the potential of PUT-based USaLT for the treatment of melanoma without the need for injection of any external agents into the body.

## Data Availability

The raw data supporting the conclusions of this article will be made available by the authors, without undue reservation.

## Supplementary material

SUPPLEMENTARY FIGURE S1 Validation of Mallory stain for muscle fibers with mouse pancreas.
