# Supplementary figures and images for: Ultrasound-assisted laser therapy for selective removal of melanoma cells
Source: Exp Biol Med (Maywood). 2024 Aug 7;249:10096. doi: 10.3389/ebm.2024.10096 (PMC11338193; doi:10.3389/ebm.2024.10096)

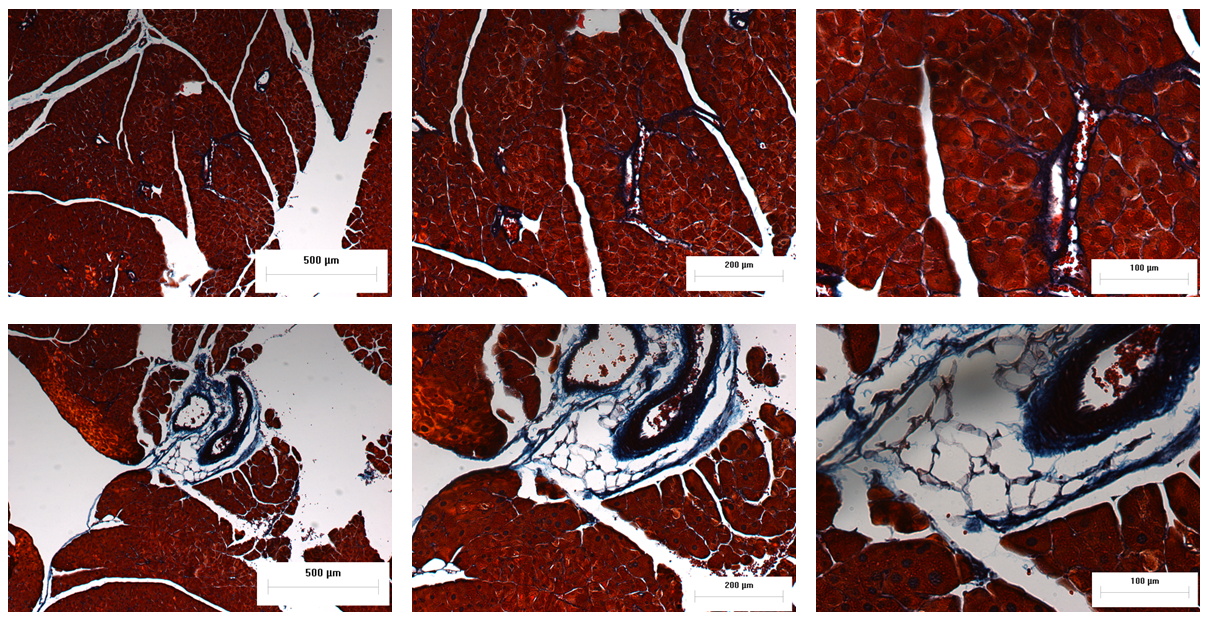

Supplement: Supplementary file 1 [file Image1.tiff]
